# Centrifugal granulation behavior in metallic powder fabrication by plasma rotating electrode process

**DOI:** 10.1038/s41598-020-75503-w

**Published:** 2020-10-28

**Authors:** Yufan Zhao, Yujie Cui, Haruko Numata, Huakang Bian, Kimio Wako, Kenta Yamanaka, Kenta Aoyagi, Akihiko Chiba

**Affiliations:** 1 Institute for Materials Research, Tohoku University, 2-1-1 Katahira, Aoba-ku, Sendai, Miyagi 980-8577 Japan; 2 Department of Metallurgy, Materials Science and Materials Processing, Graduate School of Engineering, Tohoku University, 6-6, Aramaki Aza Aoba, Aoba-ku, Sendai, Miyagi 980-8579 Japan; 3 JAMPT Corporation, 3-8, Ipponyanagi, Tagajo-shi, Yawata, Miyagi 985-0874 Japan

**Keywords:** Design, synthesis and processing, Characterization and analytical techniques

## Abstract

In recent years, spherical powders with no or minimal internal pores fabricated by the plasma rotating electrode process (PREP) have been highly recommended for powder-type additive manufacturing. Most research on PREP is aimed at establishing relationship between PREP parameters and powder size. However, almost no dedicated research on granulation behavior has been conducted so far. In the present study, PREP experiments of Ti64 and SUS316 alloys were carried out. Numerical modeling based on computational thermo-fluid dynamics was developed to analyze the granulation behavior. In particular, the roles of the additionally introduced gas blast and the morphology of the electrode end surface in fluid granulation were preliminarily investigated. The study showed that in addition to the electrode's rotating speed and diameter, manipulating the plasma arc current (i.e., the melting rate) could also be an effective way to control the PREP-powder size. According to the simulation, there were competing actions of the gas blast affecting the powder size. The gas blast created disturbance on the fluid and deepened the depression of the electrode end surface, which facilitated powder refinement. However, the cooling effect enhanced the fluid stability and hindered fluid granulation. The conclusions indicated the possibility of using various methods to manipulate PREP-powder size.

## Introduction

Metallic powders are usually applied as raw materials in various fields, including conventional powder metallurgy, injection molding, and spray coating. In recent years, additive manufacturing (AM) has become an emerging domain for metal powders^1^. Given the advances in AM, there is a growing emphasis on powder manufacturing for reliable AM-built parts.

Several techniques have been applied for metal powder fabrication. Among these, two processes—gas atomization (GA) and plasma rotating electrode process (PREP)—have been widely used to produce spherical powders for AM^2^. GA produces powders by impinging a liquid metal stream to droplets through a high-speed gas flow of nitrogen, argon, or helium. With relatively low energy consumption and a high fraction of fine powders, GA has become the most popular powder manufacturing technology for AM. However, asymmetric particles with satellites and entrapped gas pores are usually present. In contrast, PREP, which involves melting a rotating bar feedstock by a plasma, produces spherical powders that have few satellites with no or minimal internal pores. A comparative study of IN600 superalloy conducted by Chen et al.^3^ showed that the sphericity of PREP-powders was better than that of GA-powders. A survey of Ti64 powders for AM produced by different techniques conducted by Chen et al.^4^ revealed that PREP-powders (below 150 μm) possessed lower porosity than GA-powders. Such porosities of the powders can give rise to porosities in the AM-built products. Zhong et al.^5^ found that the porosity of Inconel 718 formed by laser metal deposition using PREP-powders is lower than that using GA-powders. The gas pores in raw GA-powders cannot be entirely eliminated even by hot isostatic pressing^6^. Moreover, a recent study^7^ showed that as a result of the different powder geometries, Inconel 718, built by electron beam melting using PREP powders, illustrated a broader process window than that using GA-powders. Excellent flowability, purity, and packing density are essential for even powder bed and good final quality of AM-built parts^8^. Thus, PREP-powders with high sphericity and smooth surfaces are highly recommended as feedstocks for powder-type AM^9–11^.

In PREP, the metal rod as the electrode is melted by a plasma arc induced from the cathode. Under the action of centrifugal force, the molten metal flies radially to form fine droplets and eventually solidifies into spherical particles owing to surface tension. The changes in various process parameters influence the quality and characteristics of the final powder. PREP-powders with appropriate particle size distribution (PSD) are demanded to be applied in AM because PSD determines the layer thickness, packing density, and uniform powder spreading^12,13^. The electrode rotating speed, electrode diameter, plasma arc power, etc. have a significant influence on PSD. Nie et al.^14^ found that the average powder size was roughly inversely proportional to the square root of the rotating speed. Tang et al.^15^ demonstrated that the average powder size decreased, and PSD became narrower at high rotating speeds. A similar phenomenon was observed in the studies conducted by Hsu et al.^11^ and Li et al.^16^. Liu et al.^17^ found that the average powder size decreased with increasing electrode diameter. In addition, Liu et al.^18^ showed that the melting rate (determined by arc power) significantly affected the PSD. With the increase in the melting rate, the average size of Inconel 718 powders decreased. However, Hsu et al.^11^ observed that the average size of the nitinol powders decreased by lowering the melting rate. The experimental results obtained by Liu et al.^17^ also showed that as the melting rate increased, the proportion of large-sized flake particles increased. Notably, the physical properties of molten metal is another factor in determining the PSD. Nie et al.^19^ performed PREP of different alloys, and illustrated that alloys with high surface tension and low density tended to produce coarse powder and narrow PSD.

In the industrial field, PREP is becoming a mature high-quality powder manufacturing process that has been used in commercial applications for years. It has been widely adopted in the industry that as long as a high enough rotating speed and a large enough diameter of the electrode are provided, sufficient fine powders can be produced. For example, Liu et al.^20^ developed a novel supreme-speed PREP system that could achieve a rotating speed of 28,000–36,000 rpm, which increased the yield of the fine powders (< 45 μm) compared with that of the conventional PREP. However, the challenging problems that arise in this domain are high energy consumption and manufacturing cost. Therefore, the machine cost that can satisfy the desired processing conditions is an obstacle that limits PREP-powders to be more widely used in AM^21^. To realize technological breakthroughs, careful attention should be paid to scientific principles. For PREP, understanding how the granulation behavior of the molten metal separating from the electrode under the action of centrifugal force to form droplets is the key premise of the technical optimization. To the best of our knowledge, most of the research in this field is aimed at establishing the relationship between PREP parameters and powder size, and almost no dedicated research on granulation behavior has been conducted. Thus, inadequate knowledge of fluid behavior during PREP prompted us to conduct more in-depth mechanism research.

In the present study, by varying the electrode rotating speed, electrode diameter, and plasma arc power, PREP experiments of Ti64 and SUS316 alloys were carried out. In addition to the summary of the process parameters for determining the powder size, clarification of the powder forming mechanism was the main objective. With this aim in mind, numerical modeling based on computational thermo-fluid dynamics was developed to gain insight into the granulation behavior of the molten metal during PREP. In addition, considering that the centrifugal granulation process can be accelerated by gas blast^22^, gas blast around the electrode was introduced through equipment modification. Through experiments and simulations, the mechanism of the gas blast in fluid granulation was preliminarily investigated. The role of the electrode end surface morphology in granulation behavior was also analyzed through simulations. The conclusions of this study indicate the possibility of using various methods to manipulate PREP-powder size.

## Results

### Powder size under varying process conditions

**Figure 1:**
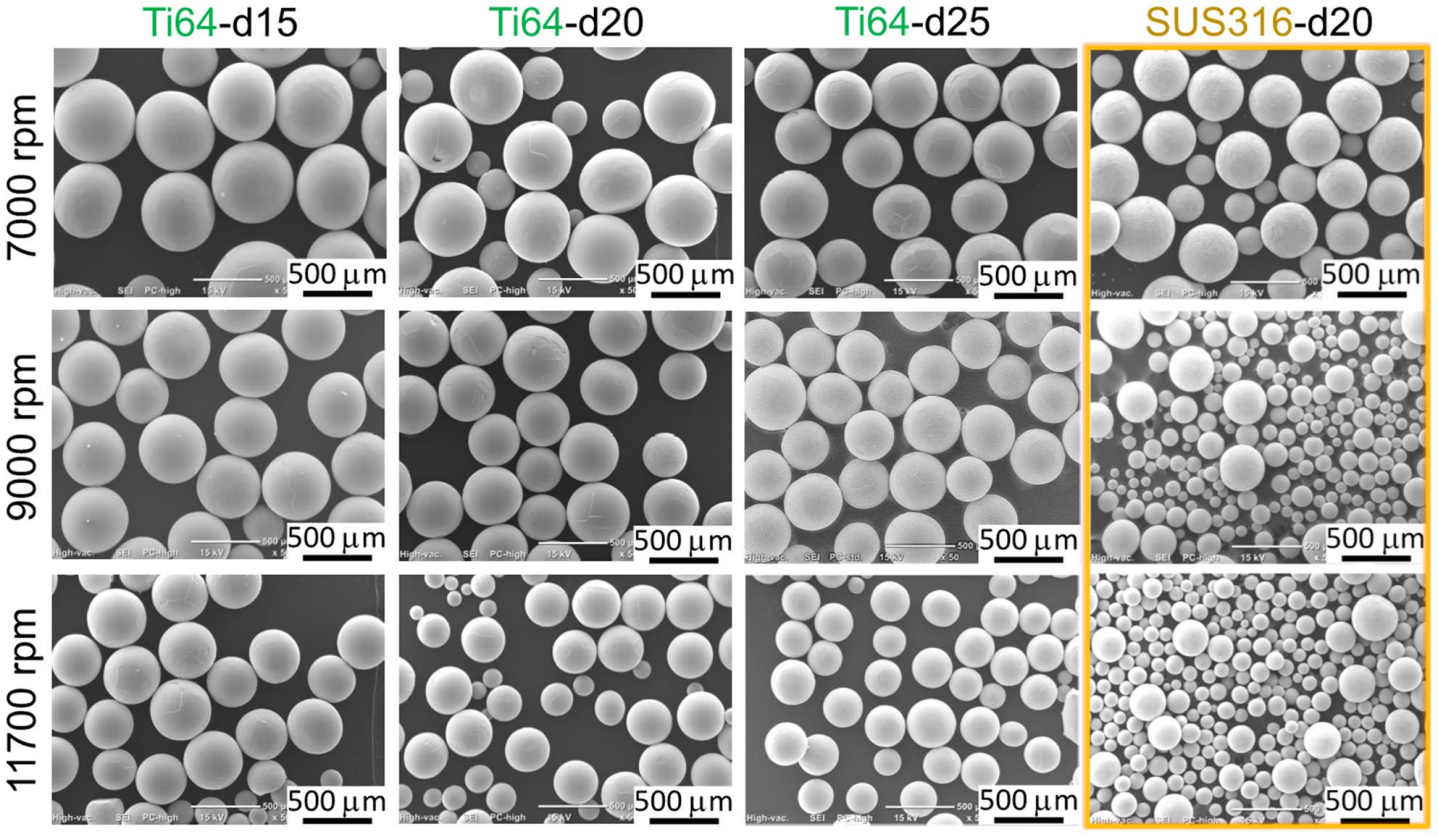
SEM images of the PREP-produced Ti64 and SUS316 powders under varying rotating speeds and electrode diameters. The arc current applied here was 80 A.

**Figure 2:**
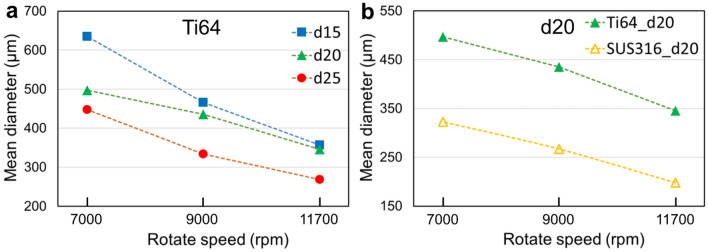
Corresponding mean diameters of the powders shown in Fig. 1.

In the experiments, the PREP operating conditions, including the rotating speed, electrode diameter, and arc current, were varied to produce Ti64 and SUS316 powders. The influence of the operating conditions and the material itself on the powder size were investigated. Figure 1 shows the scanning electron microscope (SEM) images of the PREP-produced Ti64 and SUS316 powders under varying rotating speeds and electrode diameters. The arc current applied here was 80 A. Spherical powders without satellites were obtained for both Ti64 and SUS316 under all the process conditions. The particle size distributions and mean diameters of the volume distribution (MV) were evaluated using a laser diffraction analyzer (Beckman Coulter LS230). Figure 2 shows the corresponding mean diameters of the powders. The mean diameters decreased with an increase in the rotating speed and diameter of the electrode, which was consistent with the results of some previous studies^11,14–17^. In Fig. 2b, taking the case of d20 (electrode with a diameter of 20 mm) as an example, under the same rotating speed, the mean diameter of the SUS316 alloy powder was significantly smaller than that of the Ti64 alloy. That is, in addition to the operating conditions, the physical properties of the material are also an essential factor affecting the powder size. The physical properties of Ti64 and SUS316 melt differ mainly in terms of density, viscosity, and surface tension, which are important parameters determining the centrifugal granulation behavior^19,23^.

**Figure 3:**
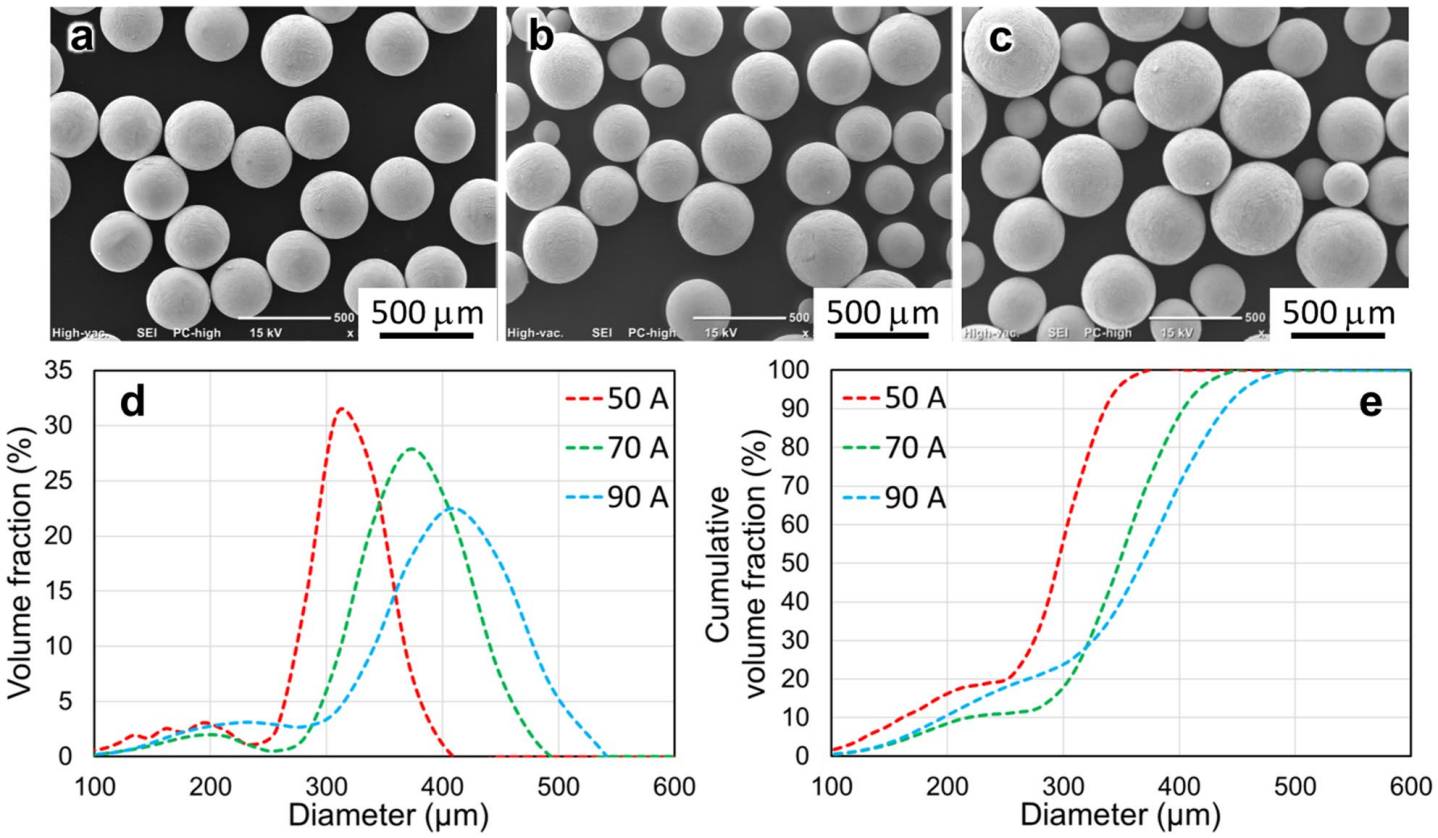
(**a**–**c**) SUS316 powders and (**d**, **e**) corresponding PSD fabricated using an electrode with a diameter of 20 mm, at 7000 rpm and currents of (**a**) 50, (**b**) 70, and (**c**) 90 A.

**Figure 4:**
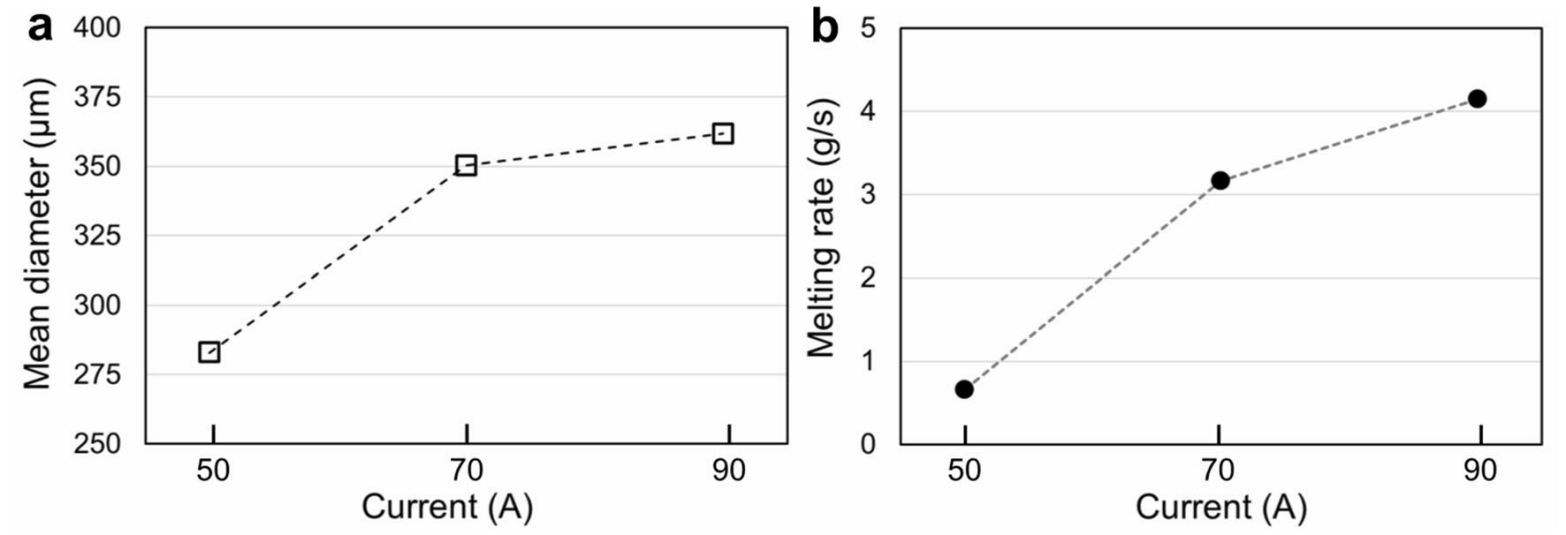
(**a**) Mean diameter of SUS316 powders increased with the increasing arc current. (**b**) The measured melting rate of SUS316 increased with the rising arc current.

In addition, the arc current had a significant effect on the powder size. Figure 3 shows the SUS316 powders fabricated using an electrode with a diameter of 20 mm, under 7000 rpm and currents of 50, 70, and 90 A. In Fig. 3d,e, the powder size and the width of the PSD increased with increasing arc current, which was similar to the results in some previous studies^11,17^. The output power of the plasma depends on the arc current, which subsequently determines the melting rate if other operating conditions are fixed. As shown in Fig. 4, the mean diameter of the SUS316 powders increased with increasing arc current, and the measured melting rate of the SUS316 increased with increasing arc current. Zhang et al.^24^ produced tin powder by centrifugal atomization and claimed that with the increase in the melt flow rate, the film thickness of the melt could be reduced before disintegration. Moreover, the fluid supply rate (melting rate) also determines the disintegration modes during the centrifugal granulation process^25–27^.

### Role of the gas blast in granulation

**Figure 5:**
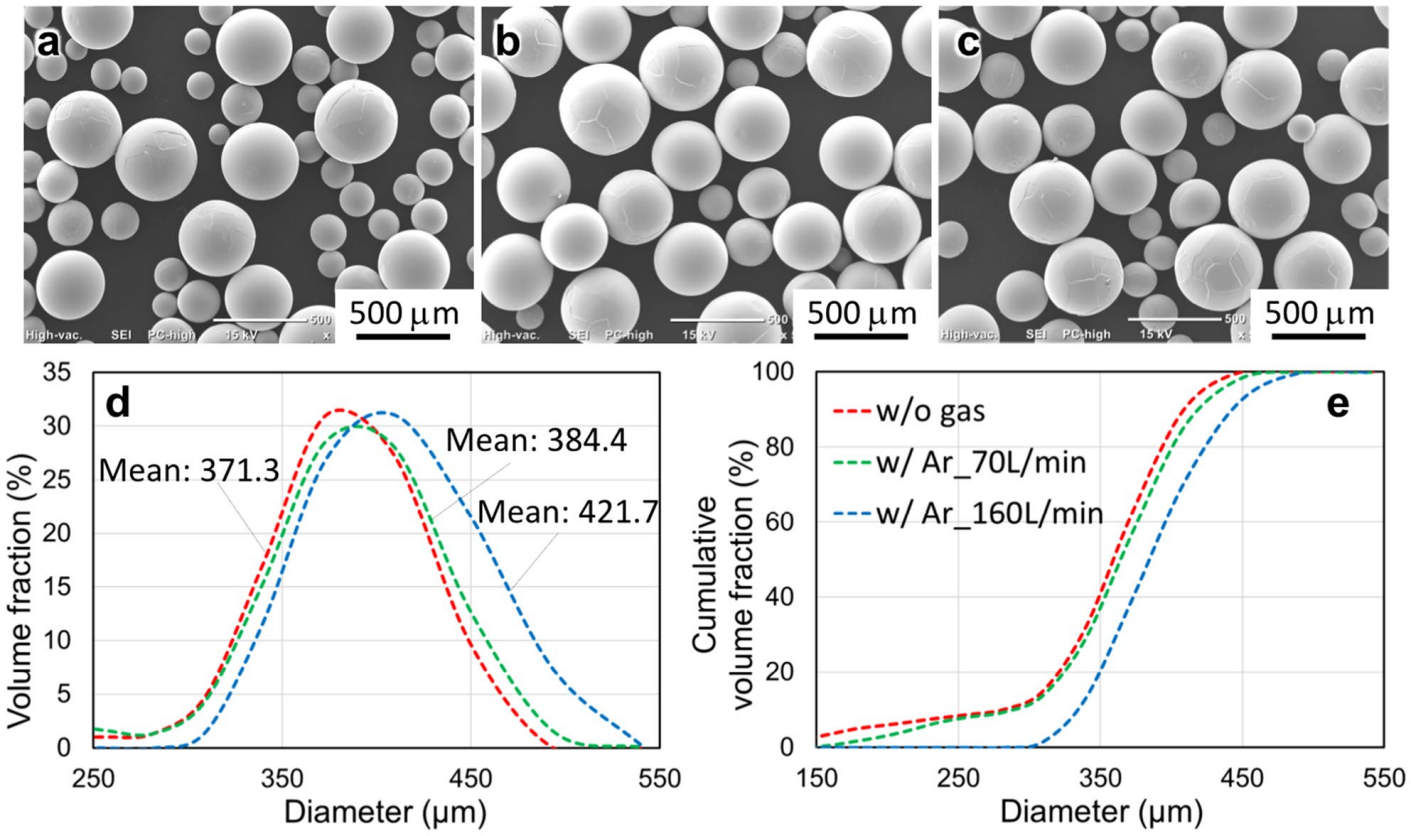
(**a**–**c**) Ti64 powders and (**d**, **e**) corresponding PSD fabricated using an electrode with a diameter of 15 mm, at 11,700 rpm and current of 80 A. The flow rates of argon were (**a**) 0, (**b**) 70 and (**c**) 160 L/min.

The application of the gas blast is assumed to promote disturbance of the fluid stream during the centrifugal granulation process. In this study, a ring-shaped outlet of the gas blast was installed around the electrode to investigate the effect of gas blast on powder size during PREP. Figure 5 shows the Ti64 powders fabricated using an electrode with a diameter of 15 mm, at 11,700 rpm and current of 80 A. The flow rates of argon were set to 0, 70, and 160 L/min. In Fig. 5d,e, the mean powder size increased as the gas flow rate increased from 0 to 160 L/min. Under the current conditions, the effect of gas blast on particle refinement was not observed. The reason might be that the physical effects of the gas blast on the fluid were manifold. On the one hand, the gas blast disturbs the fluid stream, facilitating fluid granulation and the generation of fine powders. According to previous studies on GA, the powder size decreases with increasing atomizing gas pressure^28,29^. On the other hand, the gas blast enhances the convective heat transfer between the fluid and atmosphere^30^, improving the cooling rate of the fluid pushed out of the electrode rim. Constrained cooling is accompanied by changes in the thermophysical properties of the molten metal, especially increasing the viscosity, thereby hindering the granulation process. Both effects exist and compete with each other^22,31^. The experiments in the present study indicated that, compared with the mechanical crushing of the gas blast that might promote the formation of fine powders, the cooling effect that caused a sudden change in the thermophysical properties was dominant under the current gas flow conditions. The mechanism by which the gas blast influences the centrifugal granulation behavior is analyzed using computational thermo-fluid dynamics (CtFD) simulations in the following.

**Figure 6:**
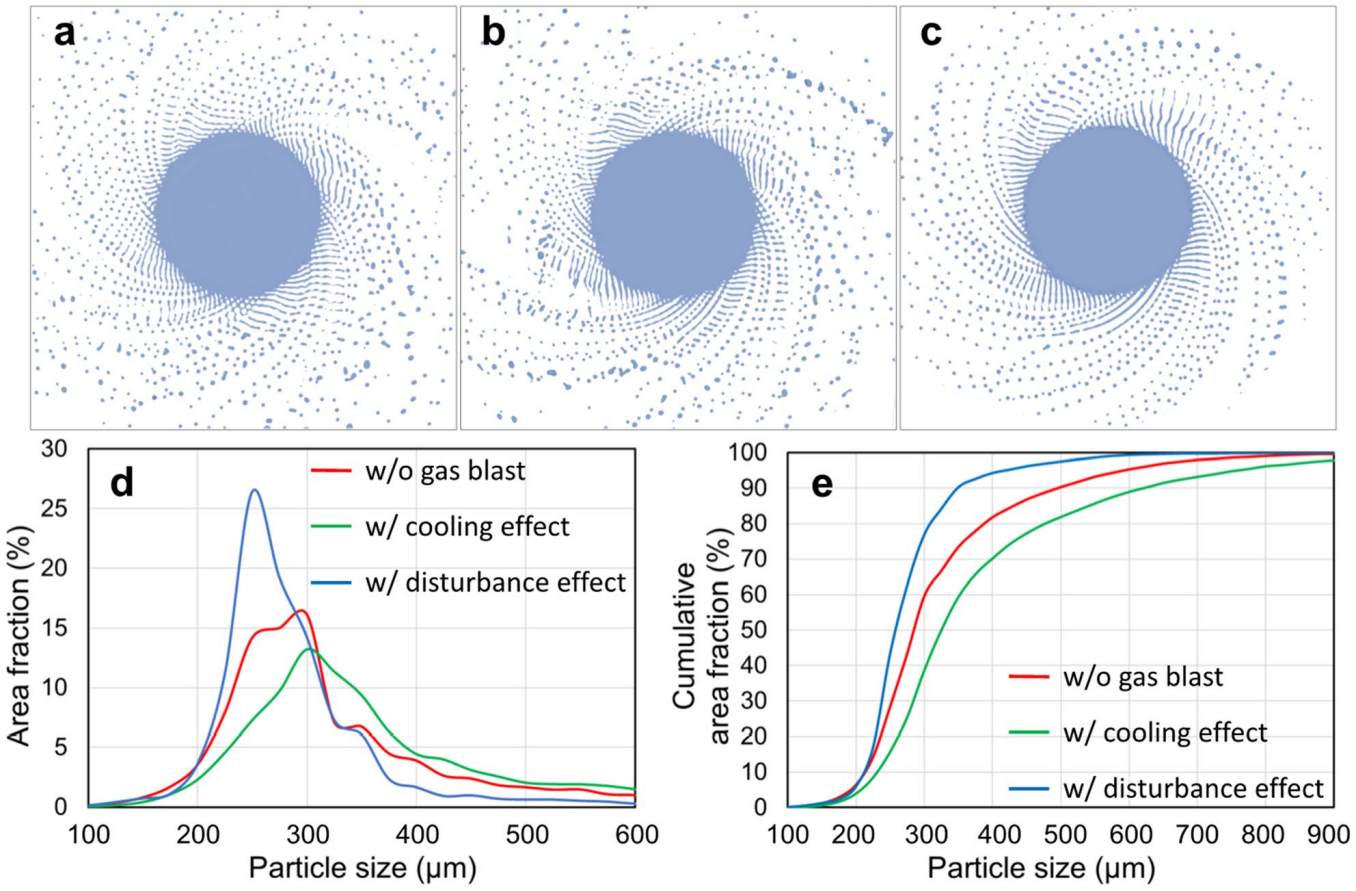
(**a**–**c**) CtFD-simulated PREP process of Ti64 and (**d**, **e**) corresponding PSD under the operating conditions corresponding to the cases in Fig. 5. The melting rate was set according to the experimentally measured value of the case shown in Fig. 5a. The simulations were performed (**a**) without gas blast, (**b**) with only cooling effect, and (**c**) with only disturbance effect.

As described previously, there are two competing actions of the gas blast in affecting powder size: the cooling effect and disturbance effect. Thus, the two effects on centrifugal granulation behavior during PREP were analyzed through CtFD simulation. Figure 6 shows the CtFD-simulated PREP process of Ti64 under the operating conditions corresponding to the cases presented in Fig. 5. The melting rate was set according to the experimentally measured value of the case shown in Fig. 5a. The simulations were performed without gas blast (Fig. 6a), with only cooling effect (Fig. 6b) and with only disturbance effect (Fig. 6c). The cooling and disturbance effects were applied by artificially increasing the heat transfer coefficient between the fluid and the ambient (ten times the original value) and by imposing an additional dynamic pressure $P_{{{\text{gas}}}}$ on the fluid surface around the electrode along the axial direction, respectively. The equivalent gas flow rate in the simulation was 200 L/min. More descriptions can be found in the Supplementary Information. The resultant powder size (Fig. 6d,e) derived from the simulation demonstrated that the cooling effect increased the powder size, while the disturbance effect helped to produce fine powders.

**Figure 7:**
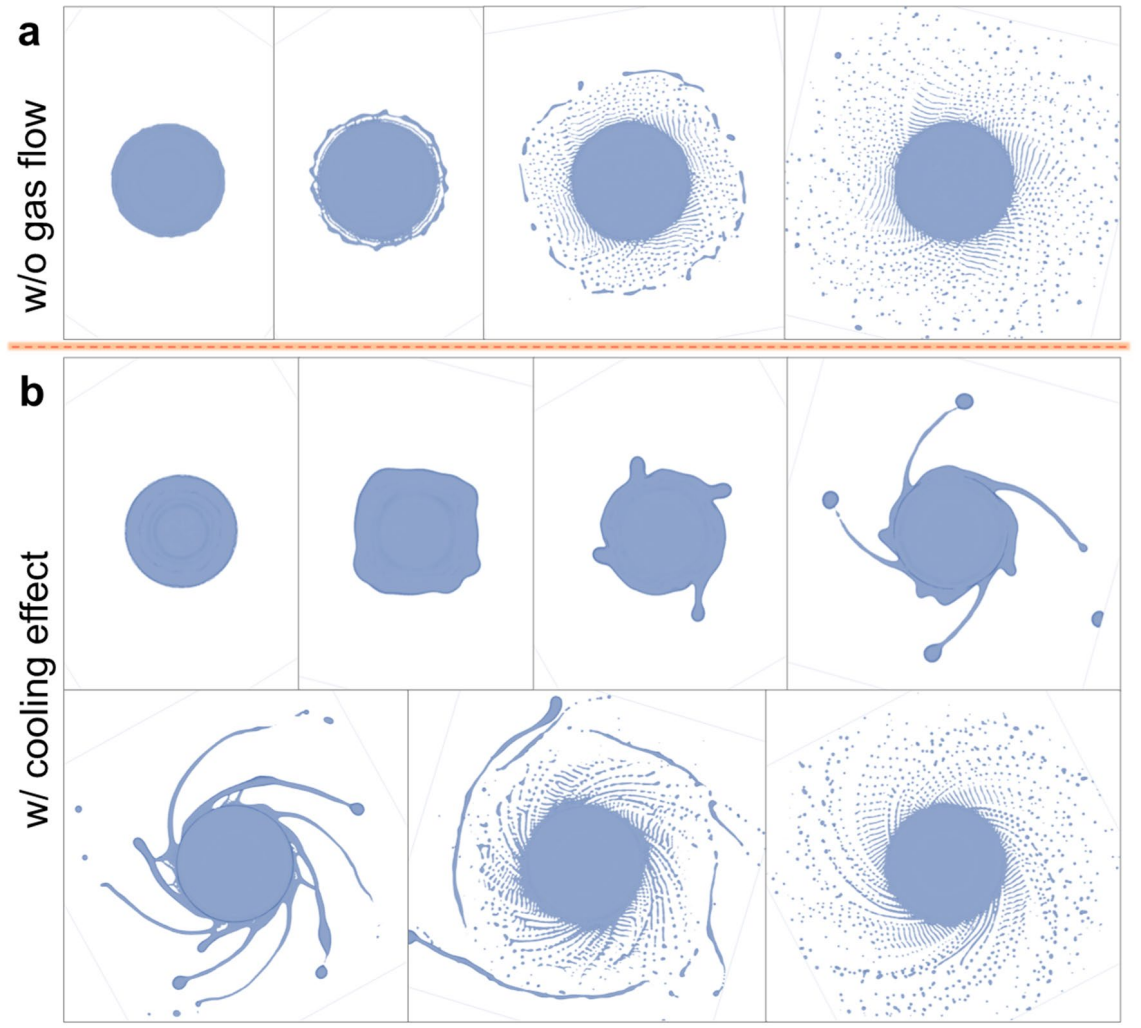
Initial stage of the centrifugal granulation process in the CtFD simulation cases (**a**) without gas blast and (**b**) with cooling effect.

The gas blast provides constrained cooling of the fluid; as a result, the temperature-dependent viscosity and surface tension of the molten metal increased. The increase in viscosity enhances the viscous force, which is an analog in fluids of the internal frictional force, hindering the fluid motion. As the surface tension results from the attraction between fluid molecules, the increase in the surface tension enhances fluid cohesion, which is not conducive to the deconstruction of the fluid and the formation of droplets. Figure 7 shows the initial stage of the centrifugal granulation process in the CtFD simulation cases without gas blast and with the cooling effect. Compared with the case without gas blast, the case with cooling effect took a long time from the start to the steady state of the granulation. As a result of high viscosity and surface tension, the fluid stability was enhanced, thereby decelerating the formation of the fluid strip/ligament. The characteristics of the typical fluid strip and ligament are shown in Fig. 15d,e. Owing to the high anti-disturbance capability, the thickness of the fluid strip/ligament and the droplet size increased. Consequently, the case with cooling effect produced the particles with a large size.

**Figure 8:**
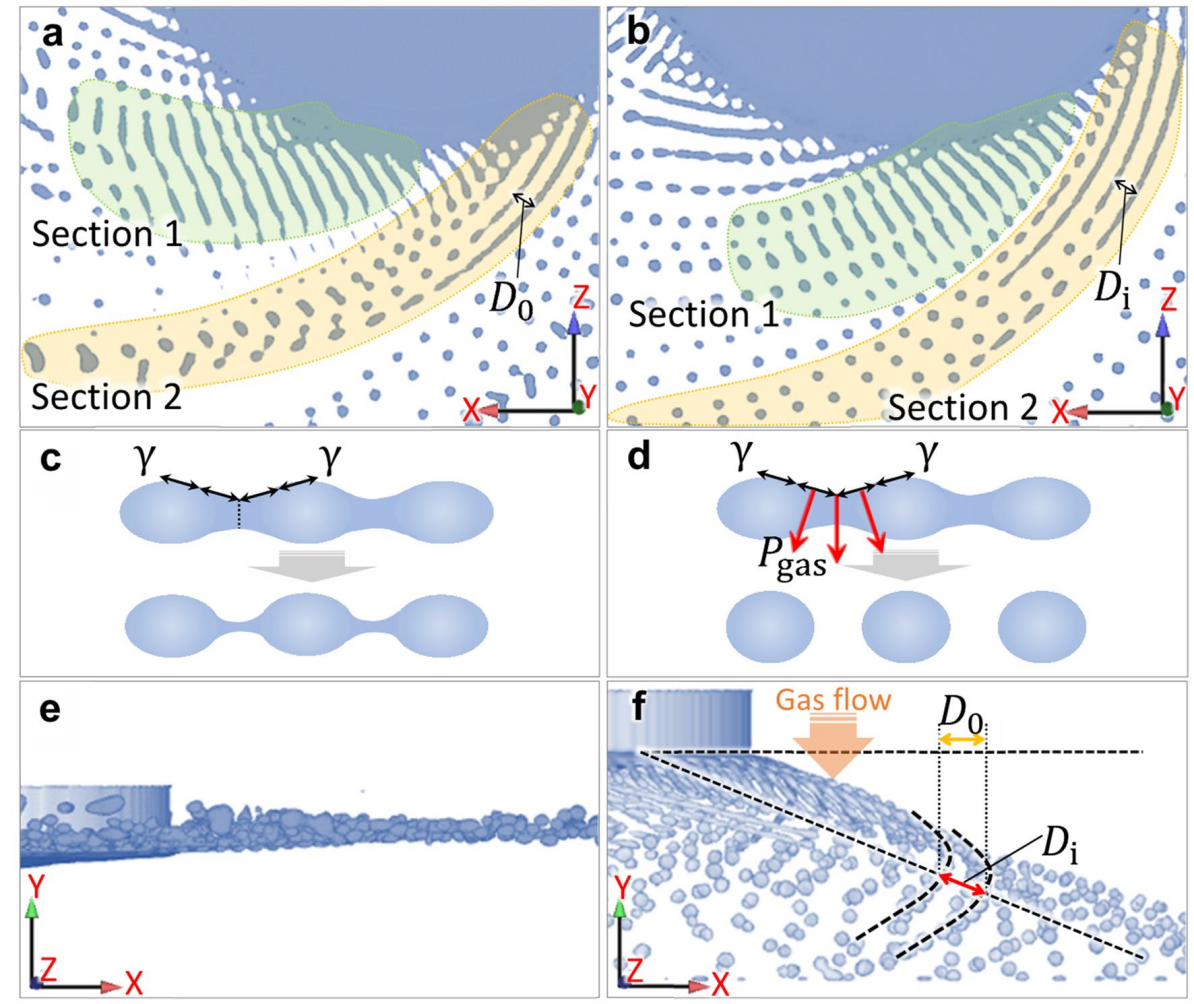
Granulation behavior in the simulation cases (**a**, **c**, **e**) without gas blast and (**b**, **d**, **f**) with disturbance effect. (**c**, **d**) Schematics show the dynamic pressure on the fluid surface exerted by the gas blast enhanced the fluid granulation. (**e**, **f**) Top views show that under the mechanical action of the gas flow, the distance between ligaments in the case with disturbance effect $D_{{\text{i}}}$ was larger than that in the case without gas blast $D_{0}$.

The gas flow also promotes the disturbance on the fluid strip/ligament owing to mechanical crushing. Figure 8 shows the granulation behavior in the simulation cases without gas blast and with disturbance effect. As shown in Fig. 8a,b, there are two feature sections that are worthy of attention. In section 1 (green), compared with the case without gas blast, the droplets were easily formed by the breakup of fluid strips in the case with disturbance effect. The schematics in Fig. 8c,d show that under the action of Rayleigh–Plateau instability and surface tension, the neck formed on the fluid strip. The dynamic pressure on the fluid surface exerted by gas blast enhanced fluid granulation, which facilitated fine powder formation in the case with disturbance effect. Moreover, in section 2 (yellow), the recombination of the droplets formed at the trailing end of the fluid ligaments occurred in the case without gas blast, which was rarely observed in the case with disturbance effect. It can be seen from the top views in Fig. 8e,f that under the mechanical action of the gas flow, the flying plane of the droplets formed an angle with the end surface of the electrode in the case with disturbance effect. As a result, the distance between ligaments in the case with the disturbance effect $D_{{\text{i}}}$ was larger than that in the case without the gas blast $D_{0}$. Subsequently, the probability of the formed droplets recombining during the later flying process was reduced, which also facilitated fine powder formation in the case with disturbance effect. That is, in addition to the enhanced disturbance, the mechanical crushing impact of the gas blast on powder refinement also contributes to the relatively non-interfering droplet trajectory.

### Role of the morphology of electrode end surface in granulation

**Figure 9:**
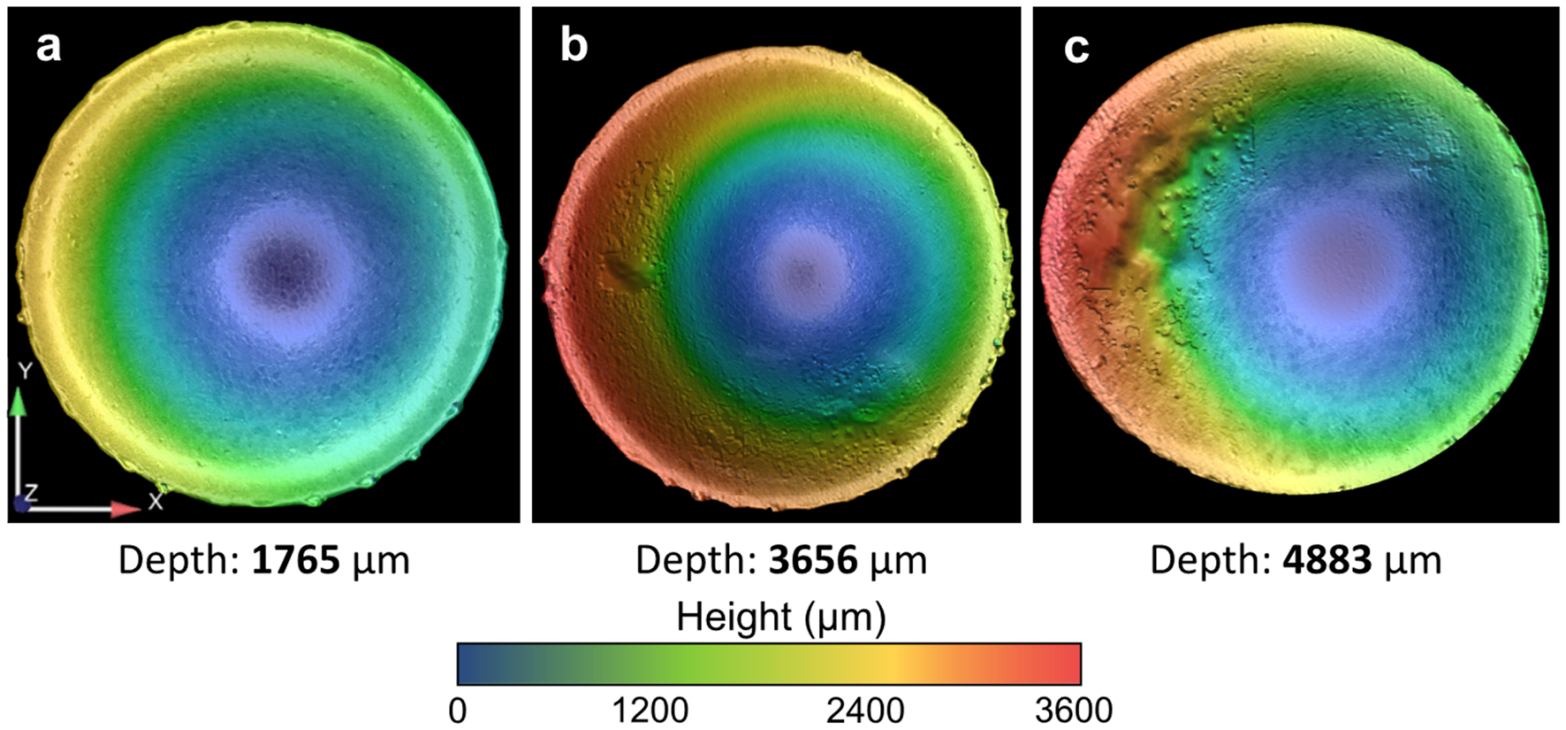
Morphology of the Ti64 electrode end surface after PREP under the operating conditions corresponding to the cases in Fig. 5 with gas flow rates of (**a**) 0, (**b**) 70, and (**c**) 160 L/min.

According to previous research on the atomization process using a rotary cup atomizer, the shape of the rotary, especially the cup depth and slope angle of the inner wall, has an significant influence on the centrifugal granulation behavior and the produced powder size^25,32^. Similarly, as shown in Fig. 9, when the operating conditions remained unchanged, the application and non-application of the gas blast changed the morphology of the electrode end surface. Compared with the case without gas blast, the depression depth of the electrode end surface was large in the case with gas blast, and the depth increased with increasing gas flow rate. As a result of the cooling effect of the gas blast around the electrode, the melting rate at the electrode center was greater than that at the electrode rim, thereby forming a depression. These depressions were slightly non-centrosymmetric, which might be caused by the uneven gas flow rate around the outer edge of the electrode; in other words, the precise control of the gas flow rate needs to be further improved.

**Figure 10:**
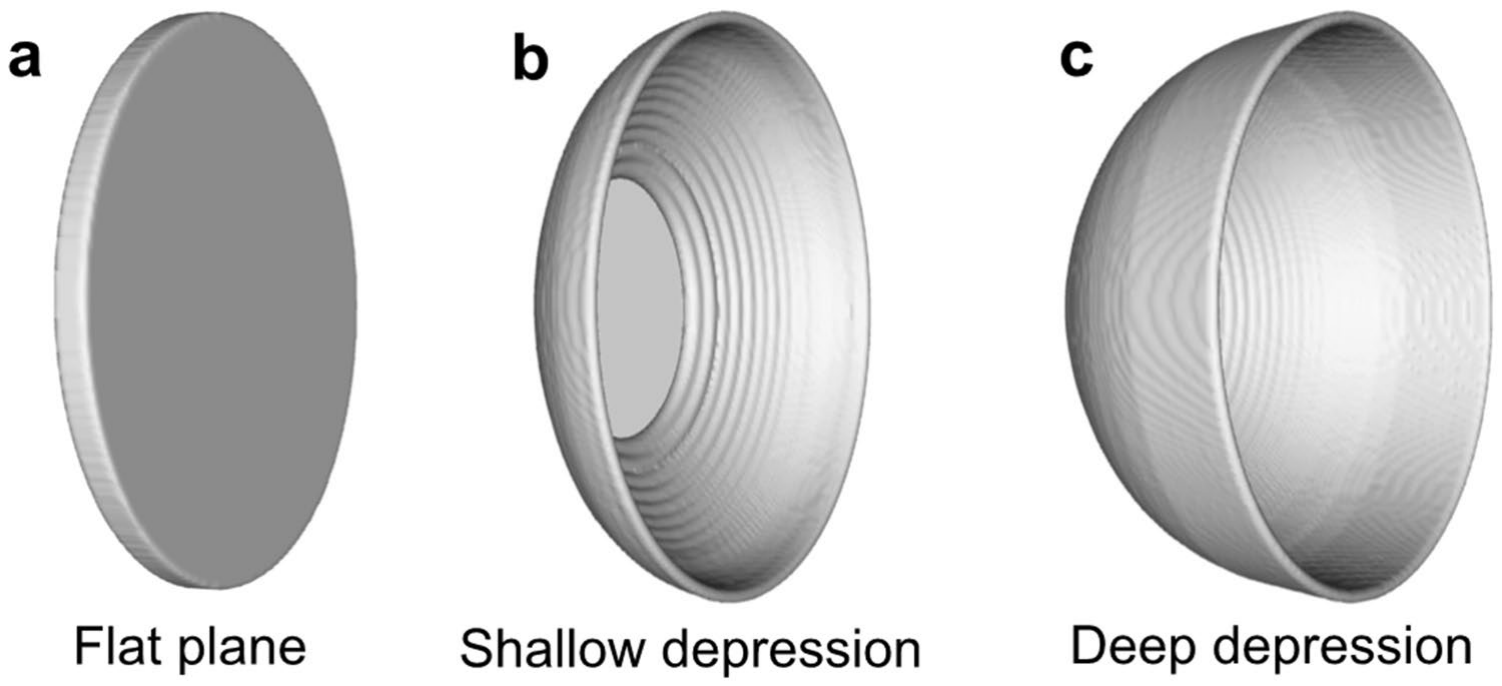
Three kinds of electrode end surfaces with the same diameter used in CtFD simulations: (**a**) flat plane, (**b**) shallow depression, and (**c**) deep depression.

**Figure 11:**
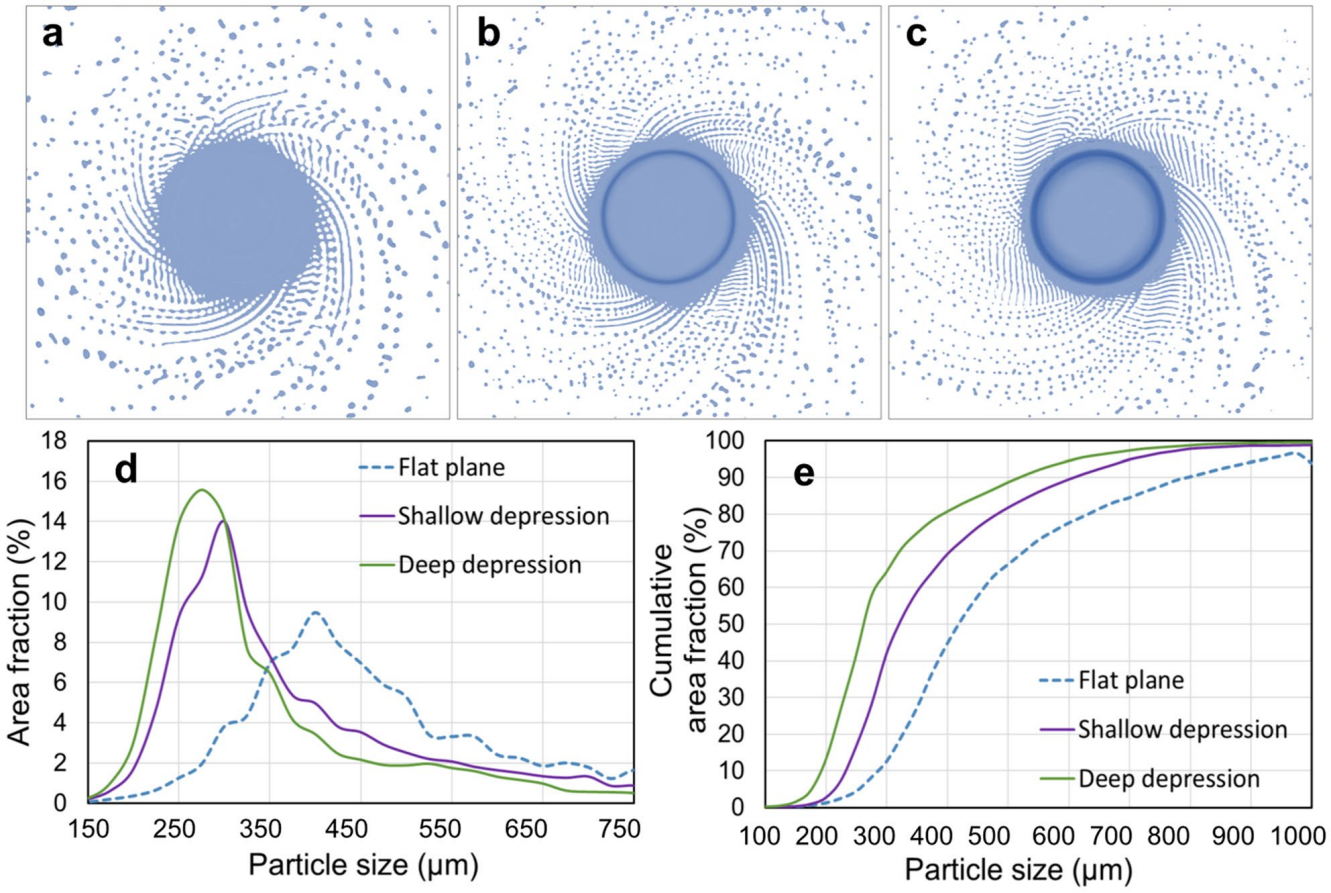
(**a**–**c**) CtFD-simulated PREP process of Ti64 and (**d**, **e**) corresponding PSD fabricated with the end surface morphology of (**a**) flat plane, (**b**) shallow depression, and (**c**) deep depression. Electron diameter was 15 mm; rotating speed was 7000 rpm; melting rate was 7.5 × 10^–7^ m^3^/s.

**Figure 12:**
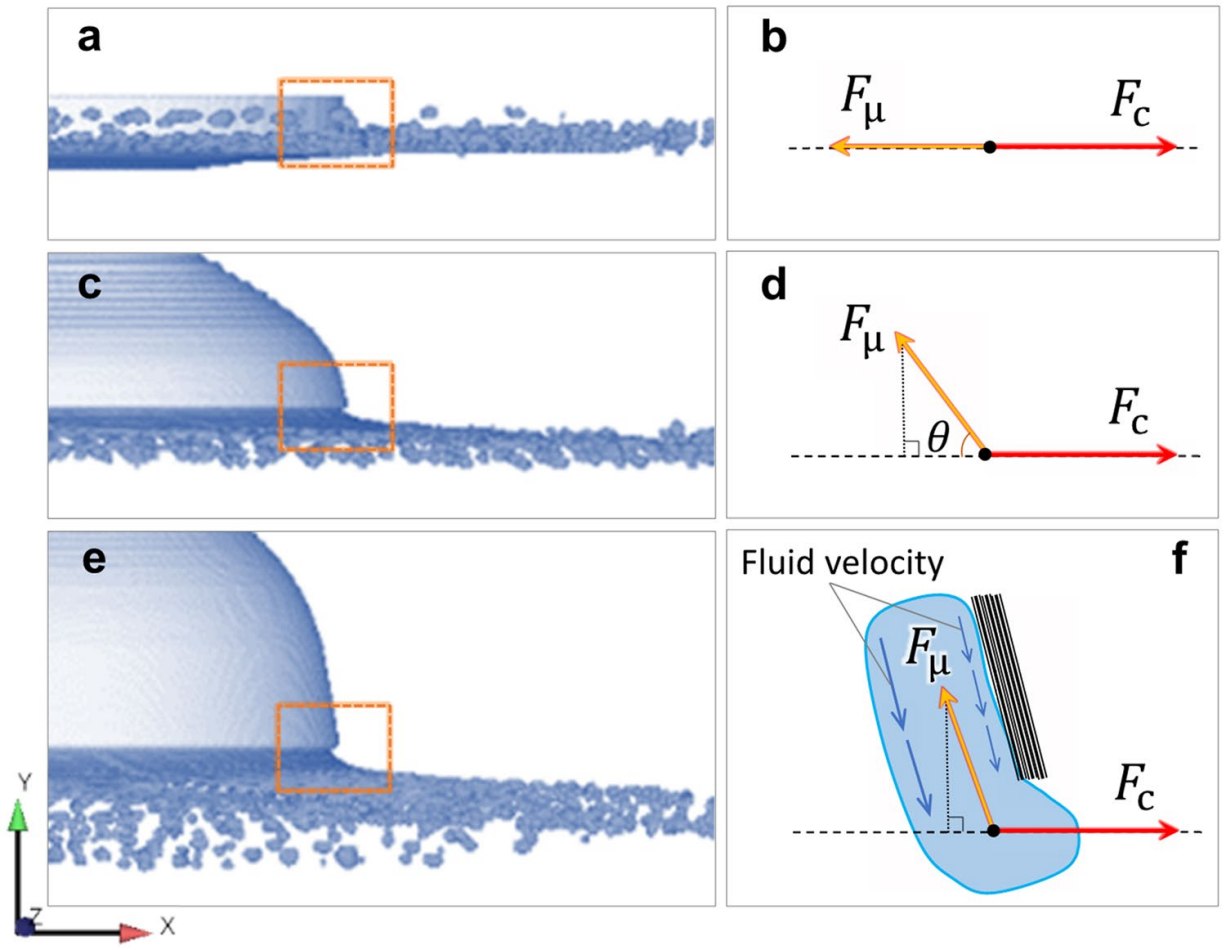
Top views of the simulation cases with the end surface morphology of (**a**, **b**) flat plane, (**c**, **d**) shallow depression, and (**e**, **f**) deep depression. Schematics show that as the depression depth increased, the angle $\theta$ increased; thus, the resultant force of the centrifugal force $F_{{\text{c}}}$ and the viscous resistance $F_{\mu }$ along the radial direction ($F_{c} - F_{\mu } \cdot \cos \theta$) was strengthened.

In order to verify the role of the electrode end surface morphology in granulation behavior during PREP, CtFD simulations were performed using three kinds of electrode end surfaces with the same diameter: flat plane (Fig. 10a), shallow depression (Fig. 10b), and deep depression (Fig. 10c). Figure 11 shows the CtFD-simulated PREP process using the three kinds of electrode end surfaces. The resultant powder size (Fig. 11d,e) derived from the simulation demonstrated that the depression of the end surface decreased the powder size, and the powder size further decreased with increasing depression depth. The fluid dynamic behavior during centrifugal granulation is determined by the integrative actions of the centrifugal force provided by the rotating electrode, and the viscous resistance originating from the viscous fluid flow on the inner wall of the depression^33^. Figure 12 shows the top views of the simulation cases with different end surface morphologies, indicating that when the fluid was pushed out from the electrode rim, the fluid motion direction formed an angle $\theta$ with respect to the radial direction of the electrode. In the schematics shown in Fig. 12b,d,f, owing to the friction between the fluid and the inner wall of the electrode depression, the fluid velocity close to the inner wall was lower than that away from the inner wall. Thus, the viscous resistance $F_{\mu }$ caused by the internal shear resistance of the fluid was parallel to the inner wall of the electrode depression. It can be seen that as the depression depth increased, the angle $\theta$ increased; thus, the resultant force of the centrifugal force $F_{{\text{c}}}$ and the viscous resistance $F_{\mu }$ along the radial direction ($F_{c} - F_{\mu } \cdot \cos \theta$) was strengthened. The large resultant force along the radial direction was beneficial for destroying fluid stability, thereby promoting the formation of fine powders. That is, the effects of disturbance and depression of the electrode end surface caused by the application of the gas blast contribute to the powder refinement, which competes with the cooling effect of the gas blast.

## Discussion

From the experimental results presented under “Powder size under varying process conditions” in the “Results” section, it can be seen that perfectly spherical PREP-powders meet the particle geometry requirements of AM in terms of powder flowability and spreading uniformity of the powder bed. Meanwhile, the powder size needs to match the predetermined layer thickness and melting capacity of the AM process. Generally, the smaller the layer thickness, the lower the energy input required, and the better the surface accuracy. Therefore, controlling the size of PREP-powders applied to AM has become the primary concern.

There is an underlying relational expression^34^ that predicts the mean diameter of powders fabricated by the centrifugal granulation process:

1 $$d_{{\text{m}}} = \frac{K}{\omega }\sqrt {\frac{\gamma }{\rho D}} ,$$

where $K$ is the correction-factor. Accordingly, a smaller powder size can be obtained at a higher rotating speed $\omega$ and using an electrode with a larger diameter $D$, which was precisely the trend of the experimental results presented under “Powder size under varying process conditions” in the “Results” section (Fig. 2a). Moreover, molten metals with low surface tension $\gamma$ and high density $\rho$ tend to produce small particles. Compared with Ti64, SUS316 has low surface tension and high density (see Supplementary Table S1). Thus, under the same operating conditions, the mean diameter of the SUS316 powder was smaller than that of the Ti64 powder (Fig. 2b). For a specific alloy, increasing the rotating speed or using a large-diameter electrode is the conventional method of manufacturing fine powders using PREP. Therefore, the corresponding energy consumption and machine cost would increase accordingly.

**Figure 13:**
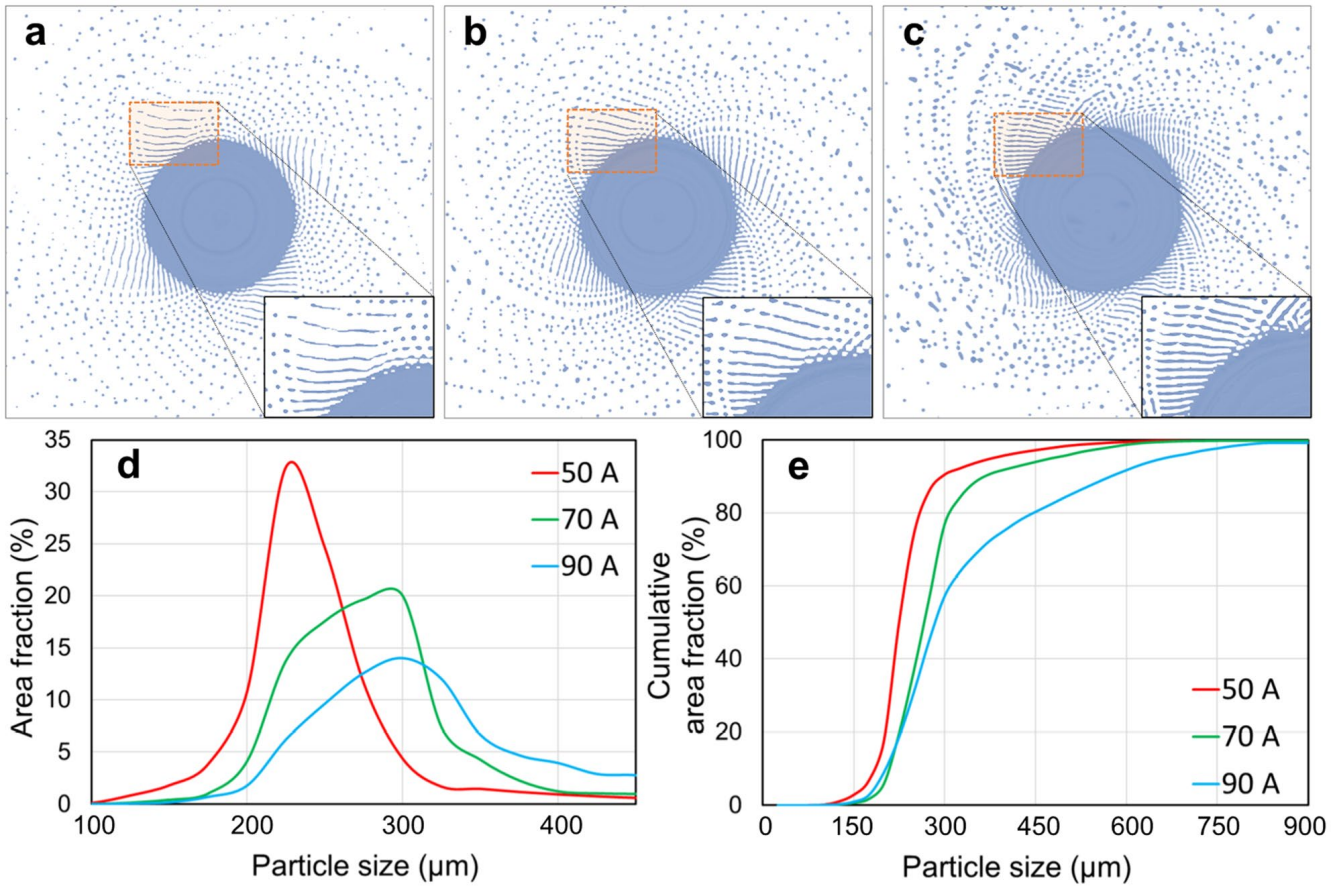
(**a**–**c**) CtFD-simulated PREP process of SUS316 and (**d**, **e**) corresponding PSD under the operating conditions corresponding to the cases in Fig. 3 with currents of (**a**) 50, (**b**) 70, and (**c**) 90 A. The melting rates determined by the currents were set according to the experimental ones shown in Fig. 4b.

Notably, in Fig. 3, when the rotating speed and diameter of the electrode remained unchanged, decreasing the arc current (reducing the melting rate) reduced the mean powder size and narrowed PSD. Figure 13 shows the CtFD-simulated PREP process of SUS316 under the operating conditions corresponding to the cases with currents of (a)50, (b)70, and (c)90 A in Fig. 3. The melting rates in the simulations were set according to the experimental results shown in Fig. 4b. By decreasing the melting rate, the powder size became smaller, and the PSD became narrower (Fig. 13d,e), which agreed with the experiments. It can be seen from the simulations that the disintegration modes were direct droplet formation (DDF) in the cases with currents of 50 and 70 A, and a transition from DDF to ligament formation (LF) in the case with a current of 90 A, which corresponded to $Hi$ 0.012, 0.056, and 0.074, respectively. In Fig. 13a–c, as the disintegration modes were mainly DDF, the powder particles were formed by the droplets that departed directly from the electrode rim and more predominantly by the deconstruction of the fluid strips. With the increasing melting rate, the thickness and spatial density of the fluid strips increased; as a result, fluid strips break up into droplets with relatively large size. Meanwhile, the small distances between the fluid strips promoted the probability that the formed droplets recombined during the later flying process. Thus, the size gap between the massive particles and the fine particles increased, that is, a wide PSD presented at a high melting rate. The experimental results and simulation analysis in the present study suggest that in addition to the rotating speed and diameter of the electrode, the melting rate $Q$ is also worthy of attention in controlling the size of PREP-powders.

## Methods

### PREP processing

**Figure 14:**
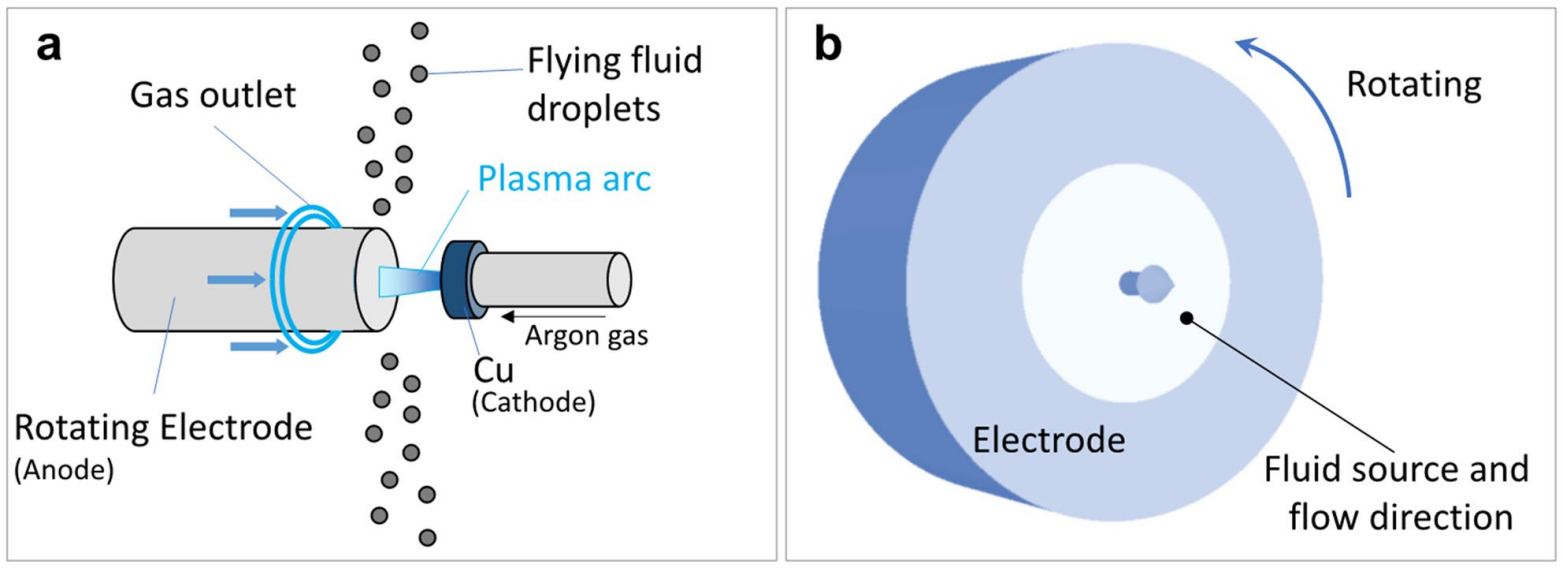
Schematic of the (**a**) PREP process and the (**b**) computational domain in CtFD simulation.

The Ti64 and SUS316 alloy powders were produced using PREP equipment made by Fuji Electronic Industrial Co., Ltd. (Tsurugashima, Saitama, Japan). Figure 14a shows a schematic of the PREP process. The powders are ejected from the high-speed rotating anode (alloy rod). The PREP chamber was filled with an inert argon atmosphere. The Ti64 and SUS316 alloy rods were provided by Aichi Steel Corp. (Tokai, Aichi, Japan). The diameters were 15 (d15), 20 (d20), and 25 mm (d25) for the Ti64 rod, and 20 mm for the SUS316 rod. The rotating speeds of the anode were set to 7000, 9000, and 11,700 rotations per minute (rpm). Plasma was generated from a copper cathode. Between the cathode and the anode, there was an argon plasma discharge of 6–9 kVA. The plasma arc currents that can determine the melting rate were set to 50, 70, 80, and 90 A. Around the anode, a ring-shaped outlet for the gas blast was additionally installed. Argon was applied as the source for plasma generation and gas blast. The flow rates of argon were set to 0, 70, and 160 L/min. The experiment under each condition and the corresponding measurements of powder size were carried out at least three times.

### Numerical simulation

During PREP, the granulation behavior of the molten alloy under the action of centrifugal force is transient and localized. Through experimental method, it is difficult to capture the dynamic characteristics of PREP. Numerical simulation based on CtFD has become a powerful tool for studying the behavior of high-velocity fluids. In this study, the 3D CtFD model was established using the software *FLOW-3D*^35^ that can solve transient scenarios with multiphysics and free surfaces of fluids.

Figure 14b shows a schematic of the computational domain that consists of a rotating rod. There was a simplification that a circular fluid source was located on the end surface of the rod, replacing the melting of the alloy with a heat source. The fluid was assigned to the temperature-dependent physical properties (molten metal). There was a void surrounding the rotating electrode at a pressure of 10^5^ Pa, which is equal to the chamber pressure. The plasma arc heats the alloy, and the instantaneous temperature of the alloy can exceed its boiling point. Therefore, the fluid source's initial temperature was set to 3000 K, which was slightly lower than the boiling point. The initial temperatures of the rotating electrode were set to 1923 K (liquidus of Ti64) for the Ti64 electrode and 1697 K (liquidus of SUS316) for the SUS316 electrode. Compared with the experimental scenario, the dimensions of the computational domain and the amount of fluid supplied (melting rate) were reduced by a ratio of 5:1. The experimental melting rate was calculated using the mass of the produced powder and the corresponding processing time. Based on the computing power of the desktop-class workstation (Intel Xeon CPU × 2 (E5-2683 v4 2.10 GHz) with 96 GB RAM), the above simplifications greatly reduce the computational burden and thus realize computing feasibility. The centrifugal granulation process can be typically depicted by three dimensionless numbers: Reynolds (Re), Ohnesorge (Oh), and Weber (We)^36^. These numbers are expressed as:

2 $$Re = \frac{4\rho Q}{{\pi \mu R}},$$

3 $$Oh = \frac{\mu }{{\sqrt {\rho \gamma R} }},$$

4 $$We = \frac{{\rho \omega^{2} R^{3} }}{\gamma },$$

where $\rho$, $\mu$ and $\gamma$ (physical properties) are the density, viscosity and surface tension of the working fluid, respectively; $Q$ and $\omega$ (operating condition) are the melting rate determined by the arc current and the rotating speed, respectively; $R$ (structural condition) is the electrode radius. If the three dimensionless numbers are the same under different processing conditions, the phenomenon characteristics of centrifugal granulation would be equivalent ^33^. Since the computational domain was scaled down compared with the experimental processing scenario, the physical properties of the fluid and the rotating speed in the simulation were adjusted accordingly. Thus, $\gamma$ and $\omega$ applied in the simulation were fit to be five and twenty-five times the experimental values, which ensured the reliability of the simulation and the correspondence with the experiment. The description of the multiphysics involved, including the fluid dynamics, heat transfer, thermophysical properties, and some coefficients, can be found in the Supplementary Information. Numerical simulations were not performed under all processing conditions like the experiments, but were performed selectively for certain conditions worth discussing and analyzing.

### Fluid disintegration mode and simulation validation

**Figure 15:**
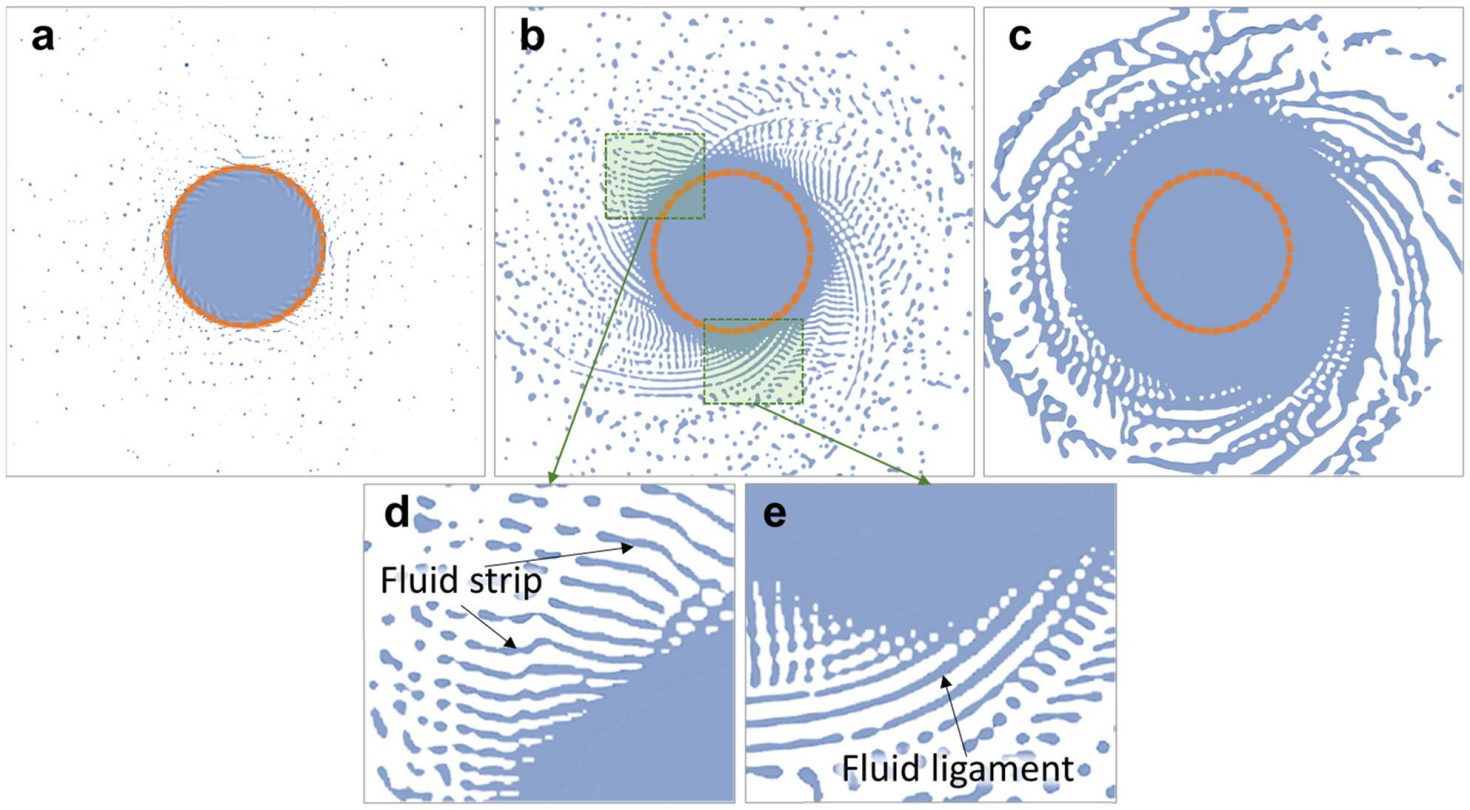
CtFD-simulated the PREP process of Ti64 with an electrode diameter of 15 mm and a rotating speed of 7000 rpm. The melting rates in the simulations were set to (**a**) 2.5 × 10^–7^, (**b**) 1 × 10^–6^, and (**c**) 7.5 × 10^–6^ m^3^/s. The corresponding disintegration modes are (**a**) DDF, (**b**) transition from DDF to LF in which (**d**) fluid strip and (**e**) ligament were observed, and (**c**) transition from LF to FF.

The disintegration mode describes how the droplets form during the centrifugal granulation process. Champagne and Angers^37^ proposed an empirical formula for the fluid granulation behavior, which predicts the transition between different modes during the rotating electrode process:

5 $$Hi = \frac{{Q\omega^{0.6} /D^{0.68} }}{{\gamma^{0.88} /\left( {\mu^{0.17} \rho^{0.71} } \right)}},$$

where the numerator is the operating conditions, including the melting rate $Q$, rotating speed $\omega$, and diameter $D$ of the electrode; the denominator is the material properties, including surface tension $\gamma$, viscosity $\mu ,$ and density $\rho$. Hinze and Milborn^38^ verified that three kinds of disintegration modes might occur during the centrifugal granulation process: DDF, LF, and film formation (FF). As $Hi$ number increases, the disintegration mode evolves from DDF to LF when $Hi$ is larger than 0.07, and from LF to FF when $Hi$ is larger than 1.33. Figure 15 shows the CtFD-simulated PREP process of Ti64 with an electrode diameter of 15 mm and a rotating speed of 7000 rpm. The melting rates in the simulations were set to 2.5 × 10^–7^ (Fig. 15a), 1 × 10^–6^ (Fig. 15b), and 7.5 × 10^–6^ m^3^/s (Fig. 15c). Thus, the resultant $Hi$ numbers were 0.0358, 0.143, and 1.08, respectively. The disintegration mode was transferred depending on the melting rate. In Fig. 15a, after flying off the electrode rim, the fluid directly breaks up into discrete droplets under the action of surface tension^23^, representing a typical DDF mode. In Fig. 15b, in addition to the strip-shaped fluid pieces (Fig. 15d) directly departed from the center fluid, arc-shaped ligaments (Fig. 15e) that were a bridge between the center fluid and droplets were observed. Owing to the Rayleigh–-Plateau instability^39^, ligaments disintegrated into droplets at trailing end, which illustrated a transition from DDF to LF. In Fig. 15c, with further increase in the melting rate, the width of the integral fluid film pushed out from the electrode rim significantly increased, and few disordered ligaments formed on the outer edge of the fluid film. The fluid broke up into large and irregularly shaped droplets, which demonstrated a transition from LF to FF. FF is usually considered to be avoided because it is not conducive to producing fine spherical powders^32^. Table 1 shows the $Hi$ numbers corresponding to the experimental conditions shown in Fig. 1. It is clear that the fluid disintegration mode of the Ti64 and SUS316 alloys was dominated by the DDF mode. The melting rates in the simulations that occurred in DDF and LF were within the range of the experimental values. The granulating evolutions observed from the CtFD simulation were basically consistent with the disintegration modes corresponding to their $Hi$ numbers. That is, the simulation conditions, including the initial temperature of the liquid phase and the setting of physical properties, were reasonable, which also validated the equivalence and reliability of the CtFD modeling.

**Table 1:** $Hi$ numbers corresponding to the experimental conditions shown in Fig. 1.

rpm	Ti64			SUS316
	d15	d20	d25	d20
11,700	0.057	0.104	0.052	0.058
9000	0.076	0.069	0.045	0.050
7000	0.059	0.068	0.038	0.069

### Experimental characterization

The powders were observed using a SEM made by JEOL Ltd. (Tokyo, Japan). A laser diffraction powder size analyzer (Beckman Coulter, Brea, California, United States) was used to measure PSD. The PSD data in the numerical simulation was derived from the 2D screenshot using Image J (National Institutes of Health, United States), which is a free image analysis software. The powder size in the simulation was expressed by the Feret diameter. Because the computational domain was scaled down compared to the experiment, the PSD results derived from the simulation were scaled up accordingly. The morphology of the electrode end surface after PREP was characterized using a Keyence VR-3200 3D measurement system (Keyence Co., Osaka, Japan).

## Conclusions

In the present study, numerical modeling based on CtFD was developed to gain insight into the molten metal granulation behavior during PREP. The mechanism of the gas blast in fluid granulation was preliminarily investigated. The role of the electrode end surface morphology in granulation behavior was also analyzed. The conclusions are summarized as follows:

1. A smaller powder size can be obtained at a higher rotating speed and using an electrode with a larger diameter. Under the same operating conditions, the size of the SUS316 powder was smaller than that of the Ti64 powder because compared to Ti64, SUS316 has low surface tension and high density.
2. In addition to the rotating speed and diameter of the electrode, manipulating the melting rate $Q$ by varying the plasma arc current can also be an effective way to control the size of PREP-powders. By decreasing the melting rate, the size of the SUS316 powders became smaller, and the PSD became narrow.
3. The simulation showed that the gas blast provided constrained cooling of the fluid; thus, the fluid stability was enhanced owing to the increase in viscosity and surface tension. Consequently, the formation of a fluid strip/ligament was decelerated. That is, the cooling effect of the gas blast produces particles with a large size.
4. The simulation showed that the gas blast provided disturbance on the fluid stream, facilitating fluid granulation and the generation of fine powders. In addition to the enhanced disturbance, the mechanical crushing effect of the gas blast on powder refinement was also attributed to the relatively non-interfering trajectory of the droplets.
5. The simulation showed that with the increasing depression depth of the electrode end surface, the resultant force of the centrifugal force and the viscous resistance along the radial direction was strengthened, which was beneficial for destroying the fluid stability, thereby promoting the formation of fine powders.

## Prospects

It can be seen that in addition to the rotating speed and diameter of the electrode, manipulating the plasma arc current (i.e., the melting rate $Q$) can also be an effective way to control the size of PREP-powders. Under the current operating conditions, the fluid disintegration mode was dominated by the DDF mode. Therefore, in other disintegration modes, especially the LF mode, the effect of the melting rate on the powder size remains unclear, which will be a future research topic.

The present study suggested that compared with the effects of disturbance and depression of the electrode end surface, the cooling effect (can coarse powder) caused by the gas blast played a dominant role in fluid granulation under the current PREP processing conditions. To limit the cooling effect but promote the disturbance effect (can refine powder) to a great extent, auxiliary equipment that can heat the gas and increase the gas flow rate is expected to be installed, thereby realizing powder refinement. Meanwhile, gas entrapment should be avoided.

By comparing the PSD results obtained from the experiment and simulation (e.g., Figs. 3d, 13d), it is clear that the simulated particle sizes were slightly smaller than the experimental ones. Although the CtFD modeling in the present study is suitable for qualitative analysis, there is still room for improvement in quantitative analysis. Specifically, further accurate measurement of fluid thermophysical properties (e.g., surface tension and viscosity), is necessary to realize quantitative analysis.

## Supplementary information

Supplementary Information.
