# Supplementary material for: Centrifugal granulation behavior in metallic powder fabrication by plasma rotating electrode process
Source: Sci Rep. 2020 Oct 28;10:18446. doi: 10.1038/s41598-020-75503-w (PMC7595198; doi:10.1038/s41598-020-75503-w)
Supplement: Supplementary file 1 — Supplementary Information. [file 41598_2020_75503_MOESM1_ESM.docx]

**Centrifugal Granulation Behavior in Metallic Powder Fabrication by Plasma Rotating Electrode Process**

Yufan Zhao^1^, Yujie Cui^1,^ *****, Haruko Numata^2^, Huakang Bian^1^, Kimio Wako^3^, Kenta Yamanaka^1^, Kenta Aoyagi^1^, and Akihiko Chiba^1^

^1^ Institute for Materials Research, Tohoku University, 2-1-1 Katahira, Aoba-ku, Sendai, Miyagi 980-8577, Japan

^2^ Gradute school of engineering, Department of Materials Processing, Tohoku University, 6-6, Aramaki Aza Aoba, Aoba-ku, Sendai, Miyagi 980-8579, Japan

^3^ JAMPT Corporation, 3-8, Ipponyanagi, Yawata, Tagajo-shi, Miyagi 985-0874, Japan

***** Corresponding author: Yujie Cui, Ph.D.; Tel.: +81 022 215 2630.
*E-mail address:* [cuiyujie@imr.tohoku.ac.jp](mailto:k.aoyagi@imr.tohoku.ac.jp).

**Supplementary information**

***Fluid flow and heat transfer in CtFD modeling***

Buoyant flow occurs owing to the varied fluid density. By introducing a temperature-dependent density, the buoyancy effect was activated. The Marangoni effect that drives the flow pattern is a result of the temperature-dependent surface tension:

| $\gamma\left( T \right)=\gamma_{L}+\frac{d\gamma}{dT}\left( T-T_{L} \right),$ | (S1) |
| --- | --- |

where $\gamma_{L}$ is the surface tension (J/m^2^) at the referenced temperature (liquidus), and ${d\gamma}/{dT}$ is the temperature coefficient of surface tension (J/m^2^∙K). Moreover, the Marangoni force, which acts along the surface tangent and drives the Marangoni convection, is expressed as

| $\frac{d\gamma}{dx}=\frac{d\gamma}{dT}\nabla T,$ | (S2) |
| --- | --- |

where $\nabla T$ is the temperature gradient (K/m) along the fluid surface. In addition to the thermal conduction *j^*^*, the heat radiation was introduced, which is expressed by the Stefan–Boltzmann law:

| $j^{*}=\varepsilon\sigma\left( T^{4}-T_{0}^{4} \right),$ | (S3) |
| --- | --- |

where $\varepsilon$ is the material emissivity, $\sigma$ is the Stefan–Boltzmann constant (W/m^2^∙K^4^), and $T_{0}$ is the ambient temperature (K). The heat transfer rate between fluid and void (ambient) $\dot{Q}$ is expressed as:

| $\dot{Q}=h_{FV}\cdot A\left( T_{F}-T_{0} \right),$ | (S4) |
| --- | --- |

where $A$ is the surface area of the fluid droplets; $h_{\mathrm{FV}}$ denotes the coefficient of heat transfer; and $T_{F}$ and $T_{0}$ are the temperatures of fluid and environment, respectively. The dynamic pressure on the fluid surface $P_{\mathrm{gas}}$ provided by the gas blast is evaluated by the following expression:

| $P_{\mathrm{gas}}=\frac{1}{2}\rho_{\mathrm{gas}}v_{\mathrm{gas}}^{2}\cos\varphi,$ | (S5) |
| --- | --- |

where $\rho_{\mathrm{gas}}$ and $v_{\mathrm{gas}}$ are the density and velocity of the blast gas, respectively. $\varphi$ is the angle between the electrode axial direction and the fluid surface normal. Physical properties and some coefficients/constants are listed in Supplementary Table S1.

| Name | Symbol and unit | Ti64 | SUS316 |
| --- | --- | --- | --- |
| Density | $\rho$ ($g/\mathrm{cm}^{3}$) | 4.42-3.75 | 7.95-6.77 |
| Viscosity | $\mu$ ($mPa\cdot s$) | 3.5-2.06 | 9-7 |
| Surface tension at $T_{L}$ | $\gamma_{L}$ ($J/{m^{2}}$) | 1.53 | 1.48 |
| Temperature coefficient of surface tension | $\frac{d\gamma}{dT}$($J/{m^{2}}\cdot K$) | -0. 00028 | -0.00019 |
| Thermal conductivity | ($W/{m\cdot K}$) | 7-34.6 | 13.4-30.5 |
| Specific heat | $C_{P}$($J/{kg\cdot K}$) | 546-831 | 470-830 |
| Emissivity | $\varepsilon$ | 0.40 | 0.66 |
| Liquidus | $T_{L}$ ($K$) | 1923 | 1697 |
| Solidus | $T_{S}$ ($K$) | 1873 | 1674 |
| Heat transfer coefficient for fluid to void | $h_{\mathrm{FV}} (W/m^{2}\cdot K)$ | 1000 | |
| Stefan-Boltzmann constant | $\sigma$ ($W/{m^{2}\cdot K^{4}}$) | 5.67e-08 | |
| Environment temperature | $T_{0}$ ($K$) | 298 | |
| Environment pressure | $P_{0}$ ($\mathrm{Mpa}$) | 0.1 | |

**Supplementary Table S1.** Physical properties of the materials and coefficients/constants applied in the simulation.
